# Supplementary material for: Diversity and Abundance of Microbial Communities in UASB Reactors during Methane Production from Hydrolyzed Wheat Straw and Lucerne
Source: Microorganisms. 2020 Sep 11;8(9):1394. doi: 10.3390/microorganisms8091394 (PMC7565072; doi:10.3390/microorganisms8091394)
Supplement: Supplementary file 1 [file microorganisms-08-01394-s001.zip › Figure S1. UASB set up.pdf]

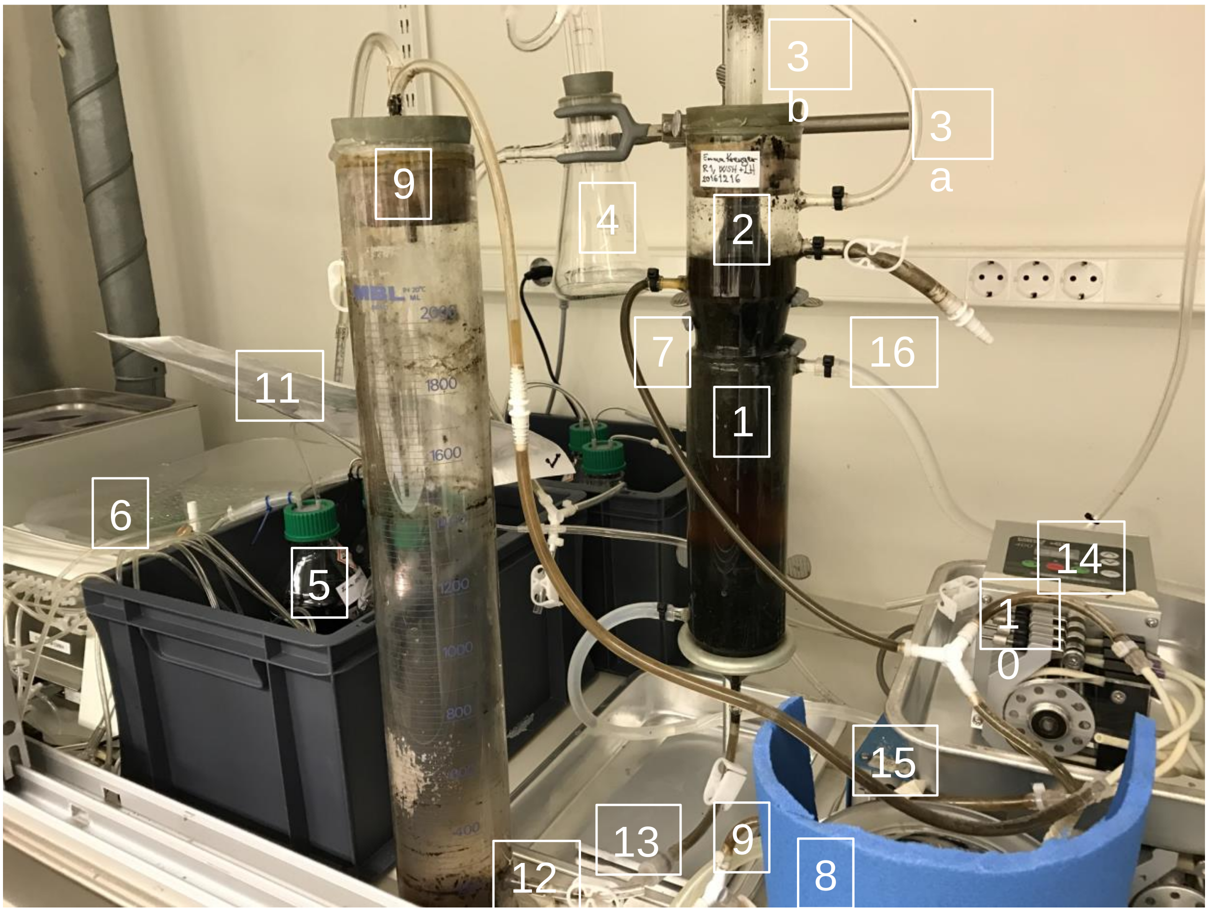

Figure S1. 1) Water-jacketed and heated up-flow anaerobic sludge blanket (UASB) reactor 0.86 L active volume. 2) Separator. 3a) Gas (from outside of separator) outlet. 3b) Gas (from inside of separator) outlet. 4) Liquid trap (in case of over-flow from the reactor). 5) Trap for CO_2 in biogas: 2x500 mL Schott bottles connected in series. Filled with 3 M NaOH and the pH indicator thymolphthalein. 6) Automatic methane potential test system (AMPTS). 7) Re-circulation from top to bottom. 8) Feed storage vessel: 500 mL Schott bottle (not shown in picture) dispersed in water in an insulated, water-jacketed and cooled vessel. Volume-equalization-balloon filled with nitrogen on top (not shown in picture). 9) Feed inlet connected to recirculation after re-circulation pump with a 3-way connector. Manual inlet at another 3-way connector just beside. 10) Effluent outlet. 11) Volume-equalization-balloon. 12) Port for removal of effluent. Removal can be done without inlet of air, by tilting the vessel when emptying. 13) Valve to hinder back flow (and reactor content loss in case of tubing-connector disruptions before this one). 14) Pump for feed and effluent. 15) Pump for recirculation. 16) Tubing with heated water, flow from bottom of water-jacket to top.
