# Supplementary material for: Diversity and Abundance of Microbial Communities in UASB Reactors during Methane Production from Hydrolyzed Wheat Straw and Lucerne
Source: Microorganisms. 2020 Sep 11;8(9):1394. doi: 10.3390/microorganisms8091394 (PMC7565072; doi:10.3390/microorganisms8091394)
Supplement: Supplementary file 1 [file microorganisms-08-01394-s001.zip › Figure S10. Acetic and propionic acids concertation dynamics predicted by ADM1.pdf]

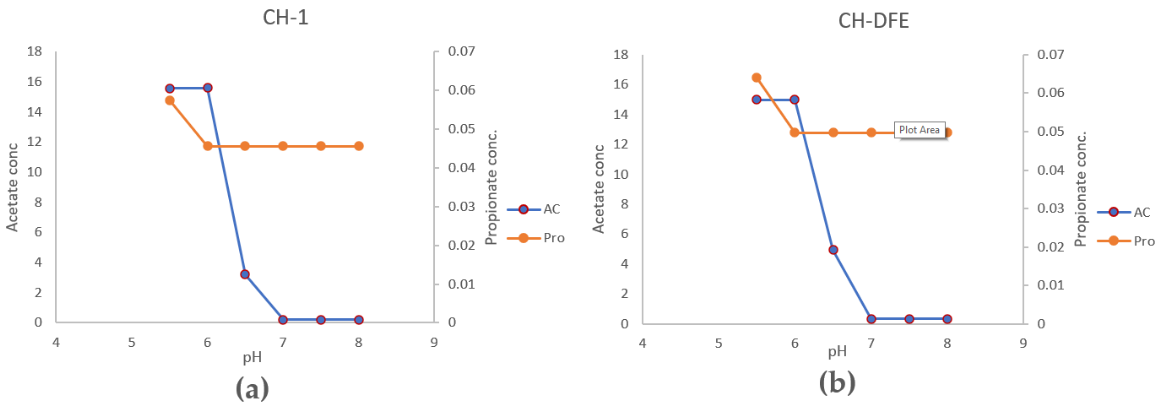

Figure S10. Changes in concentrations of acetic acid and propionic acid in UASB reactors operating with, respectively, (a) CH-1 and (b) CH-DFE as substrate, as predicted by ADM1.
