# Supplementary material for: Diversity and Abundance of Microbial Communities in UASB Reactors during Methane Production from Hydrolyzed Wheat Straw and Lucerne
Source: Microorganisms. 2020 Sep 11;8(9):1394. doi: 10.3390/microorganisms8091394 (PMC7565072; doi:10.3390/microorganisms8091394)
Supplement: Supplementary file 1 [file microorganisms-08-01394-s001.zip › Figure S2. Accumulated mass of COD fed to UASB reactors .pdf]

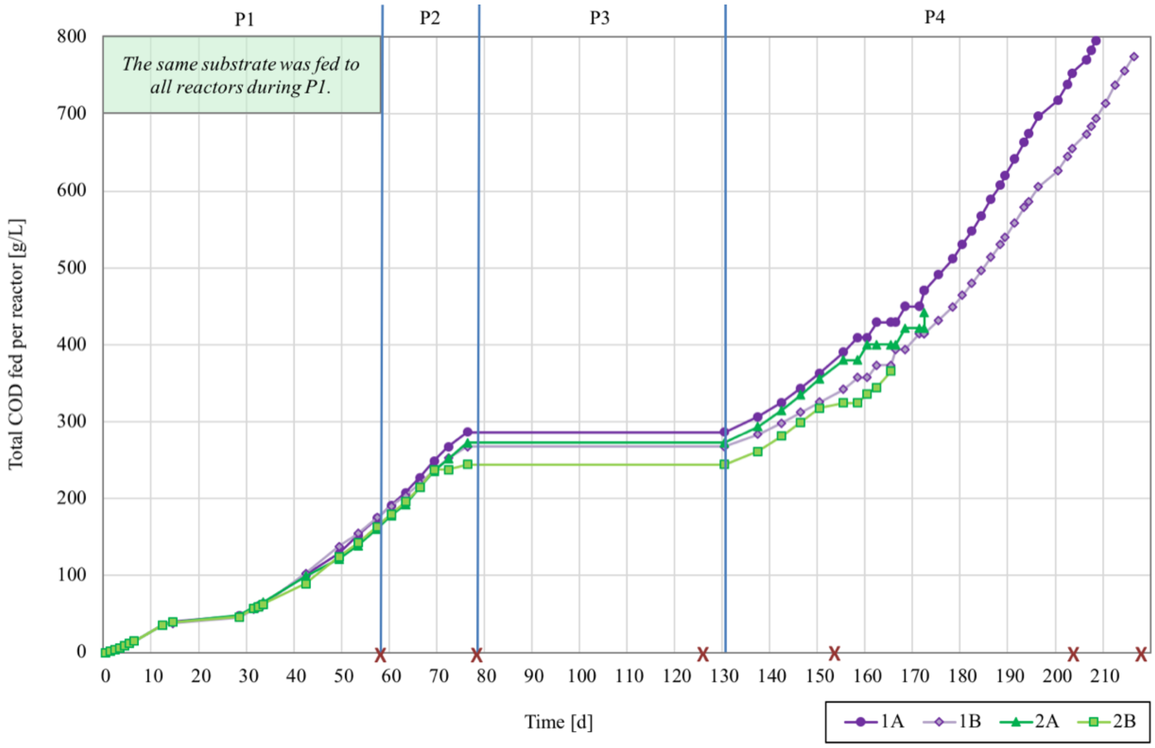

Figure S2. Total mass of COD fed to UASB reactors 1A, 1B, 2A, and 2B over time, in days (d). The blue lines indicate the changes between operating periods 1 to 4 (P1-P4), described in Table 1.
