# Supplementary material for: Diversity and Abundance of Microbial Communities in UASB Reactors during Methane Production from Hydrolyzed Wheat Straw and Lucerne
Source: Microorganisms. 2020 Sep 11;8(9):1394. doi: 10.3390/microorganisms8091394 (PMC7565072; doi:10.3390/microorganisms8091394)
Supplement: Supplementary file 1 [file microorganisms-08-01394-s001.zip › Figure S4. VFA concentrations in liquids of UASB reactors.pdf]

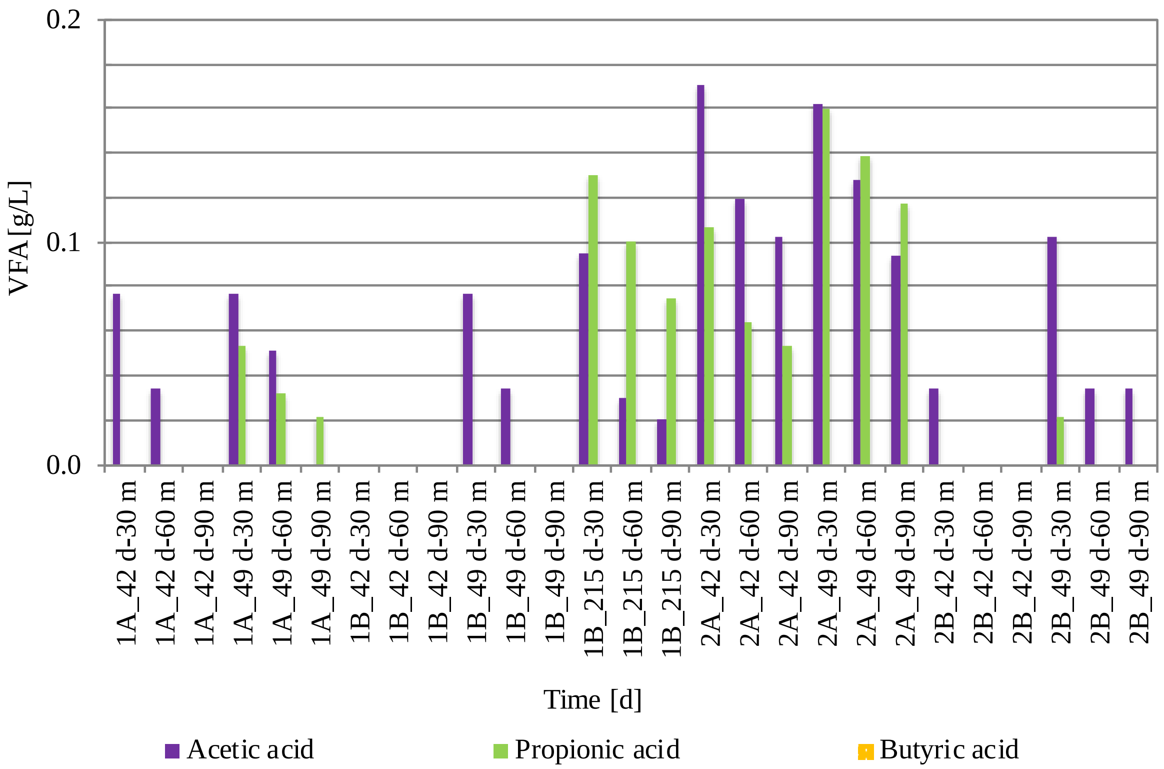

Figure S4. VFA concentrations in liquid of UASB reactors 1A, 1B, 2A, and 2B. Reactor name and time, in days (d) after start of experiment and in minutes (m) after last feeding period, are indicated on the x-axis.
