# Supplementary material for: Diversity and Abundance of Microbial Communities in UASB Reactors during Methane Production from Hydrolyzed Wheat Straw and Lucerne
Source: Microorganisms. 2020 Sep 11;8(9):1394. doi: 10.3390/microorganisms8091394 (PMC7565072; doi:10.3390/microorganisms8091394)
Supplement: Supplementary file 1 [file microorganisms-08-01394-s001.zip › Figure S5. Ratio of acids to partial alkalinity.pdf]

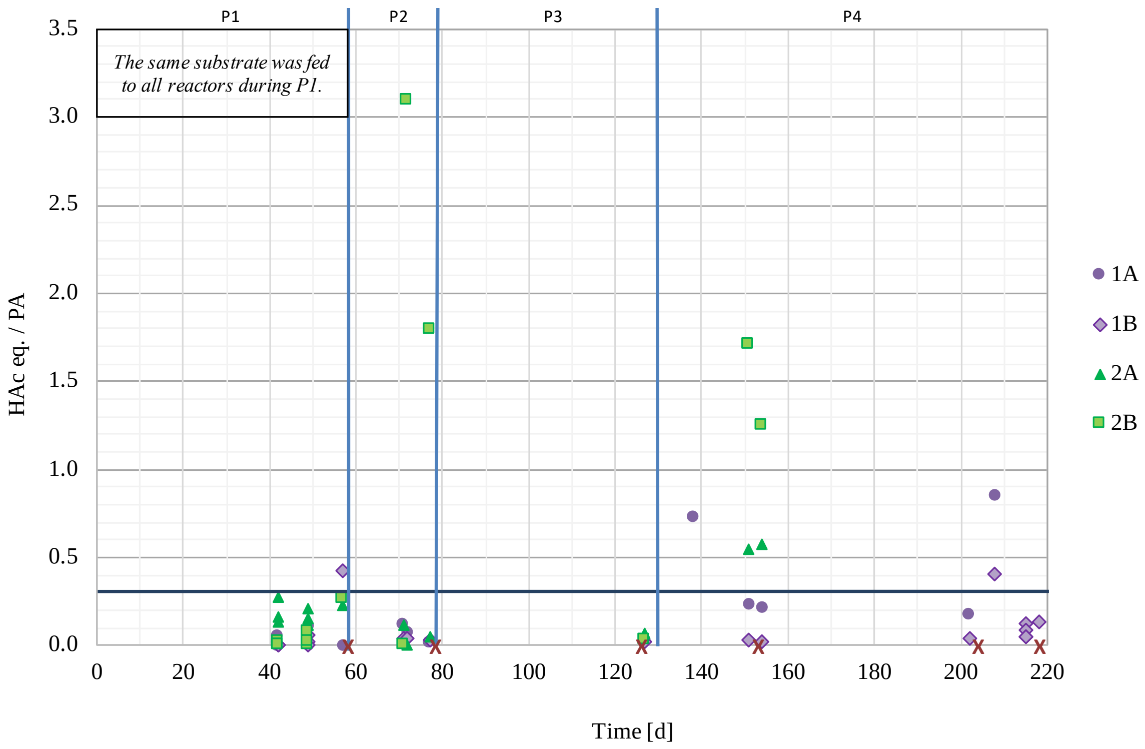

Figure S5. Ratio of acids (as g acetic acid equivalents–g HAc eq.) to partial alkalinity (PA) as g $CaCO_3$. The horizontal thick line indicates the ratio (0.3) recommended[1] as a rule of thumb to keep the intermediate alkalinity (a semiquantitative measurement of VFAs) below 0.3 for stable digestion (of chicken manure). The blue lines indicate the changes between operating periods 1 to 4 (P1-P4), described in Table 1. Time points of microbial sampling are marked with red crosses.

---

[1]Ripley, L.E.; Boyle, W.C.; Converse, J.C. Improved alkalimetric monitoring for anaerobic digestion of high-strength wastes. *Journal (Water Pollution Control Federation)* **1986**, 406-411.
