# Supplementary material for: Diversity and Abundance of Microbial Communities in UASB Reactors during Methane Production from Hydrolyzed Wheat Straw and Lucerne
Source: Microorganisms. 2020 Sep 11;8(9):1394. doi: 10.3390/microorganisms8091394 (PMC7565072; doi:10.3390/microorganisms8091394)
Supplement: Supplementary file 1 [file microorganisms-08-01394-s001.zip › Figure S8. Relative abundance of archaeal 16S rRNA genes at genus level in UASB.pdf]

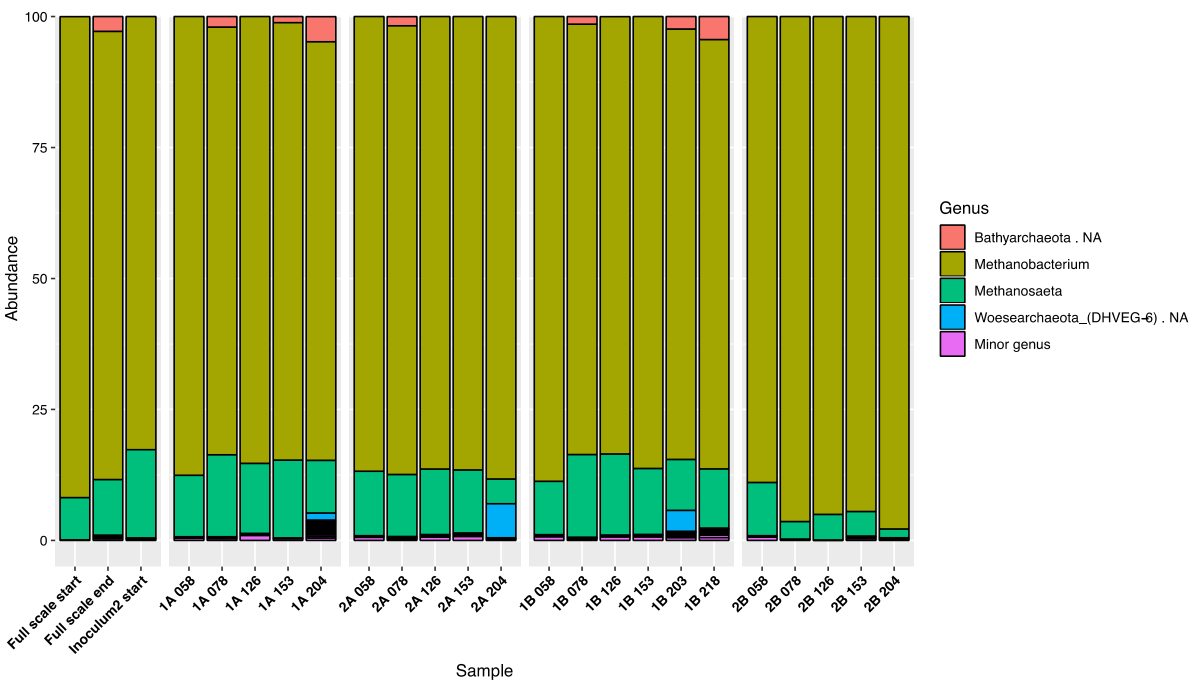

Figure S8. Relative abundance of archaeal 16S rRNA genes at genus level in UASB reactors 1A, 1B, 2A, and 2B, arranged by operation days (day 58, 78, 126, 153, 204, and additionally day 218 for 1B) and the inoculum used for reactor set-up (two types of granules, labeled 'Full scale start' and 'Inoculum2 start'), and granules from the same full-scale plant when our experiment ended, labeled 'Full scale end'. Phyla with relative abundance less than 1% were merged into 'Minor genus'. Unclassified genera are denoted by the last level of identified taxonomic name, followed by 'NA'.
