# Supplementary material for: Diversity and Abundance of Microbial Communities in UASB Reactors during Methane Production from Hydrolyzed Wheat Straw and Lucerne
Source: Microorganisms. 2020 Sep 11;8(9):1394. doi: 10.3390/microorganisms8091394 (PMC7565072; doi:10.3390/microorganisms8091394)
Supplement: Supplementary file 1 [file microorganisms-08-01394-s001.zip › Figure S9. Average log gene abundance of Order Methanobacteriales populations in the UASB.pdf]

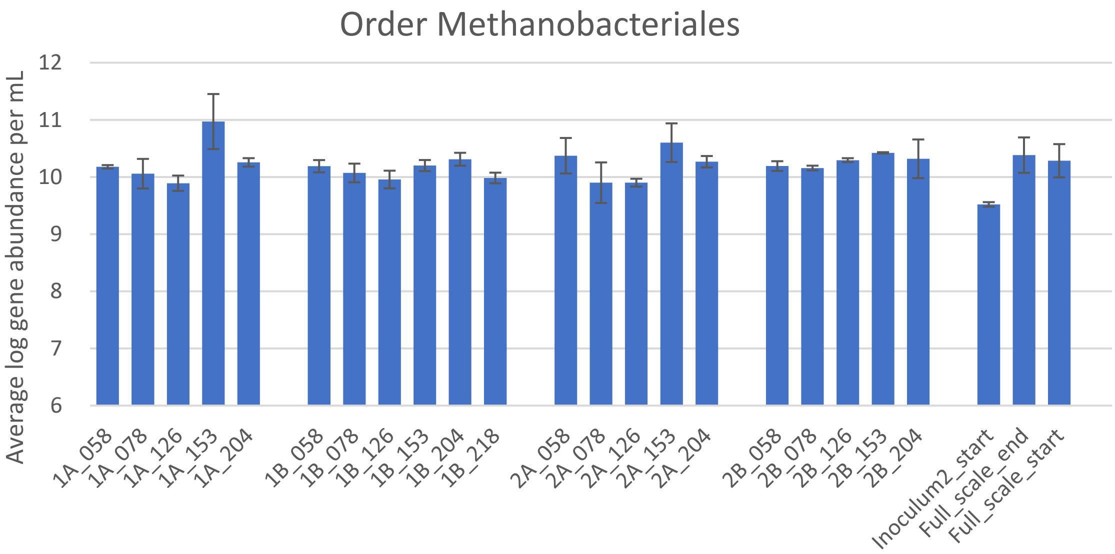

Figure S9. Average log gene abundance per mL sample obtained in qPCR analysis of Order Methanobacteriales populations in the UASB samples (1A, 1B, 2A and 2B), arranged by time (day 58, 78, 126, 153 and 204, and 218 for 1B).
