# Supplementary material for: Diversity and Abundance of Microbial Communities in UASB Reactors during Methane Production from Hydrolyzed Wheat Straw and Lucerne
Source: Microorganisms. 2020 Sep 11;8(9):1394. doi: 10.3390/microorganisms8091394 (PMC7565072; doi:10.3390/microorganisms8091394)
Supplement: Supplementary file 1 [file microorganisms-08-01394-s001.zip › Table S1. Alpha diversity of the Microbial community in UASB.pdf]

Table S1. Alpha diversity of the Microbial community in UASB reactors 1A, 1B, 2A, and 2B, arranged by operation days (day 58, 78, 126, 153, 204 and additionally day 218 for 1B) and the inoculum used for reactor set-up (two types of granules, labeled 'Full scale start' and 'Inoculum2 start'), and granules from the same full-scale plant when our experiment ended, labeled 'Full scale end'. The zero order Hill diversity (0D) of ASV reads, representing the community richness. The first order Hill diversity (1D) of ASV reads, representing the community diversity.

**Sample_name**	**0D**	**1D**
**1A_058**	388	79.4
**1A_078**	211	57.6
**1A_126**	202	39.3
**1A_153**	233	37.9
**1A_204**	214	39.5
**1B_058**	240	67.5
**1B_078**	249	55.7
**1B_126**	232	44.8
**1B_153**	273	51
**1B_204**	245	47.2
**1B_218**	208	47.8
**2A_058**	262	64
**2A_078**	218	67.6
**2A_126**	170	48.3
**2A_153**	239	53.3
**2A_204**	287	29.6
**2B_058**	238	63.1
**2B_078**	167	28.6
**2B_126**	138	24
**2B_153**	291	45.5
**2B_204**	185	22.9
**Full_scale_end**	200	43.7
**Full_scale_start**	183	37.2
**Inoculum2_start**	182	39.1
