# Supplementary material for: Diversity and Abundance of Microbial Communities in UASB Reactors during Methane Production from Hydrolyzed Wheat Straw and Lucerne
Source: Microorganisms. 2020 Sep 11;8(9):1394. doi: 10.3390/microorganisms8091394 (PMC7565072; doi:10.3390/microorganisms8091394)
Supplement: Supplementary file 1 [file microorganisms-08-01394-s001.zip › Table S2. Alpha diversity of the Archaeal community in UASB.pdf]

Table S2. Alpha diversity of the Archaeal community in UASB reactors 1A, 1B, 2A, and 2B, arranged by operation days (day 58, 78, 126, 153, 204 and additionally day 218 for 1B) and the inoculum used for reactor set-up (two types of granules, labeled 'Full scale start' and 'Inoculum2 start'), and granules from the same full-scale plant when our experiment ended, labeled 'Full scale end'. The zero order Hill diversity (0D) of ASV reads, representing the community richness. The first order Hill diversity (1D) of ASV reads, representing the community diversity.

Sample_name	0D	1D
**1A_058**	27	8.2
**1A_078**	30.3	6.8
**1A_126**	24.3	6.5
**1A_153**	26.3	5.8
**1A_204**	71.3	8.6
**1B_058**	26.3	7.9
**1B_078**	34.3	6.8
**1B_126**	30.7	6.4
**1B_153**	27	5.7
**1B_204**	54.7	5.3
**1B_218**	62.7	5.3
**2A_058**	31.7	7.2
**2A_078**	41.3	6.7
**2A_126**	36.7	6.7
**2A_153**	34.7	6
**2A_204**	37.3	3.2
**2B_058**	27.3	7.4
**2B_078**	23	5.8
**2B_126**	25	5.7
**2B_153**	30.3	4.6
**2B_204**	27.7	3.1
**Full_scale_end**	47.3	6.9
**Full_scale_start**	22	9.1
**Inoculum2_start**	17.3	4.4
